# Three-Dimensional Innate Mobility of the Human Foot on Coronally-Wedged Surfaces Using a Biplane X-Ray Fluoroscopy

**DOI:** 10.3389/fbioe.2022.800572

**Published:** 2022-02-04

**Authors:** Takuo Negishi, Shuhei Nozaki, Kohta Ito, Hiroyuki Seki, Koh Hosoda, Takeo Nagura, Nobuaki Imanishi, Masahiro Jinzaki, Naomichi Ogihara

**Affiliations:** ^1^ Department of Biological Sciences, Graduate School of Science, The University of Tokyo, Tokyo, Japan; ^2^ Graduate School of Human Sciences, Osaka University, Suita, Japan; ^3^ Department of Orthopedic Surgery, Ogikubo Hospital, Tokyo, Japan; ^4^ Graduate School of Engineering Science, Osaka University, Suita, Japan; ^5^ Department of Clinical Biomechanics, Keio University School of Medicine, Tokyo, Japan; ^6^ Department of Anatomy, Keio University School of Medicine, Tokyo, Japan; ^7^ Department of Radiology, Keio University School of Medicine, Tokyo, Japan

**Keywords:** foot kinematics, tibio-calcaneal coupling, bipedal locomotion, human evolution, insole, subtalar joint, ankle ligamentous sprain, knee osteoarthritis

## Abstract

Improving our understanding on how the foot and ankle joints kinematically adapt to coronally wedged surfaces is important for clarifying the pathogenetic mechanism and possible interventions for the treatment and prevention of foot and lower leg injuries. It is also crucial to interpret the basic biomechanics and functions of the human foot that evolved as an adaptation to obligatory bipedal locomotion. Therefore, we investigated the three-dimensional (3D) bone kinematics of human cadaver feet on level (0°, LS), medially wedged (−10°, MWS), and laterally wedged (+10°, LWS) surfaces under axial loading using a biplanar X-ray fluoroscopy system. Five healthy cadaver feet were axially loaded up to 60 kg (588N) and biplanar fluoroscopic images of the foot and ankle were acquired during axial loading. For the 3D visualization and quantification of detailed foot bony movements, a model-based registration method was employed. The results indicated that the human foot was more largely deformed from the natural posture when the foot was placed on the MWS than on the LWS. During the process of human evolution, the human foot may have retained the ability to more flexibly invert as in African apes to better conform to MWS, possibly because this ability was more adaptive even for terrestrial locomotion on uneven terrains. Moreover, the talus and tibia were externally rotated when the foot was placed on the MWS due to the inversion of the calcaneus, and they were internally rotated when the foot was placed on the LWS due to the eversion of the calcaneus, owing to the structurally embedded mobility of the human talocalcaneal joint. Deformation of the foot during axial loading was relatively smaller on the MWS due to restricted eversion of the calcaneus. The present study provided new insights about kinematic adaptation of the human foot to coronally wedged surfaces that is inherently embedded and prescribed in its anatomical structure. Such detailed descriptions may increase our understanding of the pathogenetic mechanism and possible interventions for the treatment and prevention of foot and lower leg injuries, as well as the evolution of the human foot.

## Introduction

During walking and running, the rearfoot starts to evert (pronate) as the heel contacts the ground (Wright et al., 1964; Nawoczenski et al., 1998; Stacoff et al., 2000; Pohl et al., 2007; Behling et al., 2020), such that the body weight can be supported by the ball of the hallux, allowing effective push-off by the great toe in the late stance phase. However, if the rearfoot is excessively pronated or supinated during walking and running, the risk of injury is potentially increased, leading to foot and knee pathology (Gross et al., 2011; Tong and Kong, 2013; Eizentals et al., 2019; Mei et al., 2019; Nigg et al., 2019; Dempster et al., 2021). For example, excessive pronation of the rearfoot is known to result in larger internal rotation of the tibia due to the mechanical coupling of the foot and leg (Lapidus, 1963; Olerud and Rosendahl, 1987; Hintermann et al., 1994). This so-called tibio-calcaneal coupling movement has been associated with knee or lower leg injuries (Hintermann and Nigg, 1998) such as medial tibial stress syndrome, patellofemoral pain, and knee osteoarthritis (Neal et al., 2014; Almeheyawi et al., 2021). Moreover, excessive supination of the foot has been linked to ankle sprains because a higher ankle joint moment may be applied in the supination direction owing to the supinated posture of the foot, causing ligamentous sprains of the lateral side of the ankle, namely the anterior talofibular and calcaneofibular ligaments (Fong et al., 2009).

Foot orthoses such as lateral or medial wedge insole (wedge inclined towards the outside and inside of the foot, respectively) are often prescribed for the treatment and prevention of foot and leg injuries. The medial wedge insole is used to reduce excessive foot pronation and tibial internal rotation, to control aching pain associated with flatfoot deformity characterized by increased varus alignment of the foot (Braga et al., 2019) and ligamentous sprain of the anterior talofibular ligament (Tochigi, 2003). The lateral wedge insole is used for knee osteoarthritis to laterally shift the position of the center of pressure to reduce the knee adduction moment during walking (Reeves and Bowling, 2011). Detailed information about how the alignment of the calcaneus and talus with respect to the tibia on the coronal plane affects the 3D mobility and hence the mechanics of the ankle and foot joint is also vital for improved results of total ankle arthroplasty (Michael et al., 2008; Frigg et al., 2010). Improving our understanding on how the ankle and foot joint complex kinematically adapt to coronally wedged surfaces is important for clarifying the pathogenetic mechanism and possible interventions for the treatment and prevention of foot and lower leg injuries.

Understanding the kinematics of the ankle and foot joint complex is also critical for clarifying the evolution of the human foot, which is highly specialized to adapt to obligatory bipedal locomotion during the course of human evolution. The feet of African great apes such as chimpanzees, bonobos, and gorillas are known to be inverted with respect to the tibia as an adaptation to vertical climbing. The inverted foot allows African great apes to position their foot plantar surface against the side of the vertical substrate during climbing (DeSilva, 2009) to facilitate grasping by increasing the contact surface area of the foot to the substrate. In contrast, the human foot is more perpendicularly oriented with respect to the tibia, because the relatively everted foot of humans is more adapted to terrestrial locomotion by allowing the tibia to vault over the foot in a straight line along the longitudinal axis of the foot during the stance phase of gait. Detailed analysis of the kinematics of the ankle and foot joint complex on coronally wedged surfaces may help clarify the evolution and basic biomechanics of structurally embedded functions of the human foot that evolved as adaptations to obligatory bipedal locomotion.

The human foot is a complex system formed by compound articulations of the tibia, fibula, talus, and calcaneus (talocrural, subtalar, and tibiofibular joints), which function as a unit. Therefore, previous studies have attempted to investigate foot kinematics using a multi-segment foot model (Damavandi et al., 2010; Segal et al., 2018) and kinetics using force sensors (Urry, 2002; Dixon and Pearsall, 2010; Damavandi et al., 2012; Segal et al., 2018) while walking on coronally wedged surfaces. However, surface markers are less reliable for capturing bone movements owing to skin movement artifacts (Reinschmidt et al., 1997; Westblad et al., 2002; Nester et al., 2007). Therefore, the radiographic technique was previously used to measure 3D bone kinematics of the foot during walking (e.g., Koo et al., 2015; Balsdon et al., 2016; Campbell et al., 2016) and running (e.g., Peltz et al., 2014; Hoffman et al., 2015; Welte et al., 2021) on level surface. As for the eversion and inversion of the foot on coronally wedged surfaces, Lundberg et al. (1989) used a biplane X-ray system to capture three-dimensional (3D) motions of the foot bones of subjects standing on one foot on a coronally wedged platform *in vivo*. More recently, Fernández et al. (2020) used a standing computed tomography scanner to capture the feet of subjects standing on coronally wedged surfaces *in vivo*. However, in these studies, the foot bone movements were generated as a consequence of postural adjustment and balance control by the trunk and leg muscles, including those of the foot. Therefore, it is difficult to infer the innate (physiological) mobility of the foot based on the morphology and structure of the human foot. In contrast, if cadaver feet are used, the effects of muscle activation and force distribution of the plantar surface of the foot can be better controlled.

Consequently, in the present study, the 3D movements of the foot bones were quantified under axial loading *in vitro* using biplane fluoroscopy, with the aim of developing a foundational understanding of the innate mobility of the bones in the human foot on coronally wedged surfaces that are inherently embedded and prescribed in their morphology and structure. Specifically, we tested if the 3D mobility of the foot bones due to axial loading differ between on level and coronally wedged surfaces. Although 3D kinematics of the cadaver feet on coronally wedged surfaces during axial loading have been reported previously (Olerud and Rosendahl, 1987; Hintermann et al., 1994), these studies did not attempt to capture the 3D movements of the talus, navicular, and cuboid of the foot on coronally wedged surfaces during axial loading to clarify the innate mobility of the human foot. The present study offers new insights about kinematic adaptation of the human foot to coronally wedged surfaces that is inherently embedded and prescribed in its anatomical structure.

## Materials and Methods

### Specimens

Five fresh frozen cadaver lower legs of humans (average age at death, 80 years; range, 63–92 years; four males and one female) were used in this study. All specimens were donated to the Keio University School of Medicine with the consent of the families of all donors. The present study was approved by the ethics committee of the School of Medicine and the Faculty of Science and Technology, Keio University, and by the Office for Life Science Research Ethics and Safety, The University of Tokyo. All methods were performed in accordance with the relevant guidelines and regulations. Visual and radiographic inspections were performed to confirm that all specimens were free of foot and ankle pathologies. The specimens were cut at the middle of the shank, and the extrinsic foot muscles were completely stripped and removed from the shafts of the tibia and fibula.

### Kinematic Measurement

**FIGURE 1:**
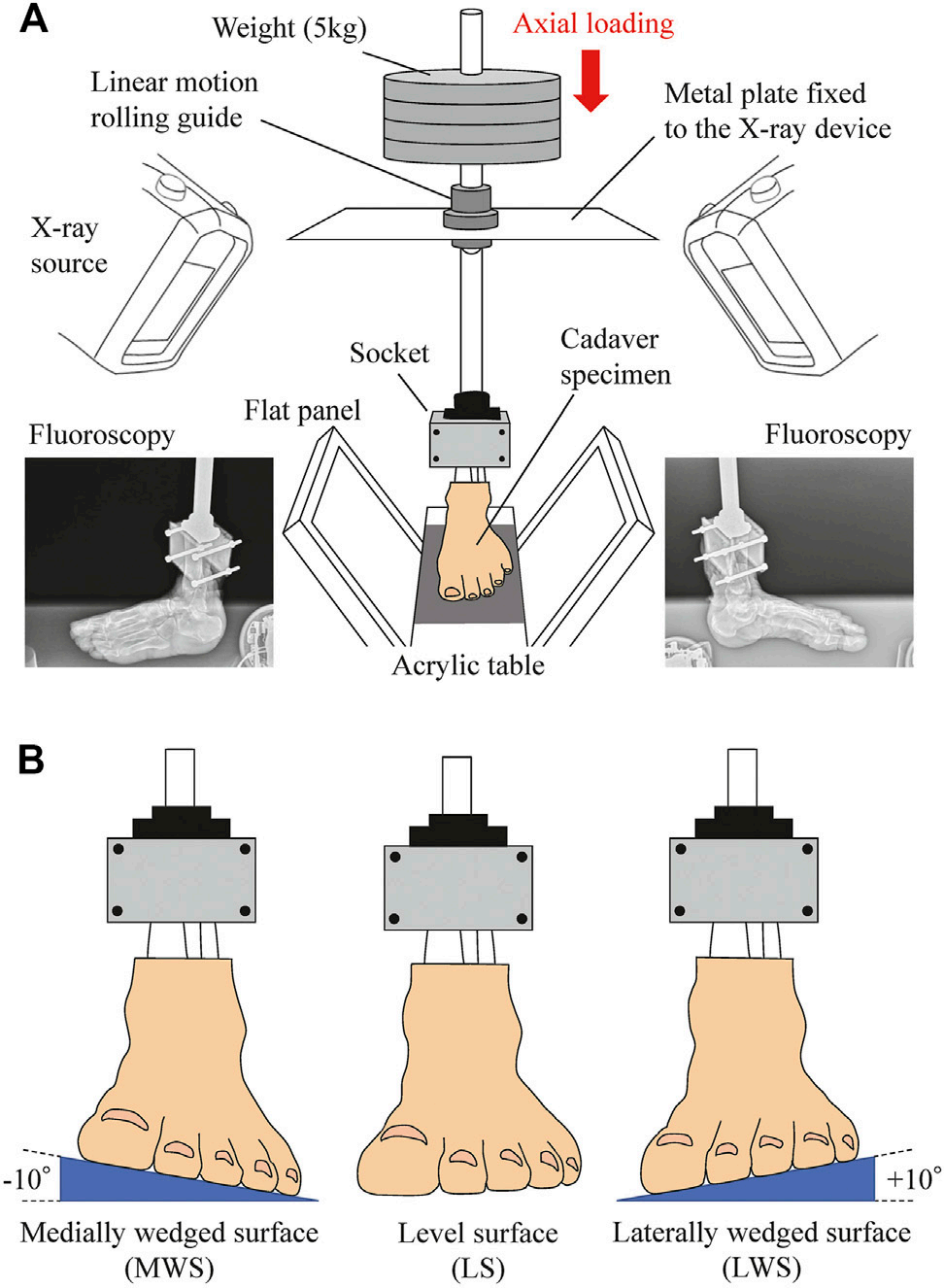
Experimental setup to measure 3D kinematics of the foot on level and coronally-wedged surfaces using biplane fluoroscopy. **(A)** The biplane X-ray fluoroscopic system consists of two X-ray sources and corresponding detector panels positioned in a quasi-orthogonal arrangement. The specimen was fixed to the shaft using a 3D-printed socket (mold). The proximal end of the lower limb was sandwiched by the anterior and posterior molds and tightly screwed to an aluminum holder that was connected in line with the shaft. The shaft goes through a linear motion rolling guide fixed to the X-ray system, such that it can freely move along and rotate around the vertical axis, allowing unrestrained tibial rotation, occurring due to the tibio-calcaneal coupling generated during eversion and inversion of the foot. **(B)** Cadaver feet were placed on LS (0°), MWS (−10°), and LWS (+10°).

In the present study, the 3D movements of cadaver foot bones were measured using a custom-made biplane fluoroscopy system and a model matching method, as described in detail elsewhere (Ito et al., 2015; Ito et al., 2017). Briefly, the cadaver feet placed on level (0°, LS), medially wedged (−10°, MWS), and laterally wedged (+10°, LWS) surfaces were loaded vertically up to 60 kg (588 N, approximately equal to average body mass of Japanese elderly) from the zero-loading condition (only the 3.3-kg vertical shaft was fixed), and the 3D kinematics of the foot bones were captured using a biplanar fluoroscopy system (Figure 1). The wedge angle of 10° was selected to avoid unexpected damage of cadaver specimens due to axial loading on the wedged surfaces. The surfaces were made of hard styroform, so that virtually no deformation of the surface occurred due to axial loading. The feet did not slip during axial loading because of slightly rough surface of the styrofoam. 3D bone surface models of the cadaver foot were generated using computed tomography prior to the experiment. The models were then registered to the fluoroscopic images to quantify the 3D translations and orientations of the foot bones [calcaneus, talus, navicular, cuboid, and five metatarsals (MTs)], and the tibia due to axial loading.

**FIGURE 2:**
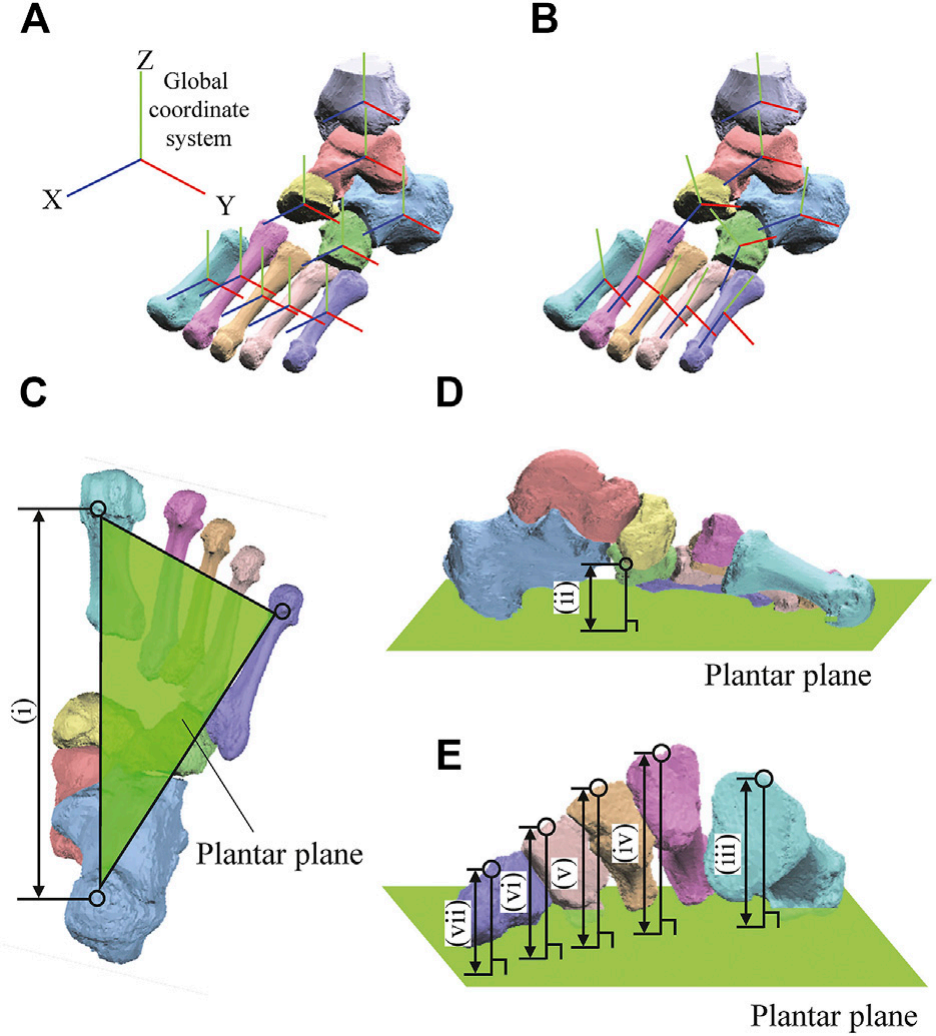
Definitions of the coordinate systems **(A,B)** and foot dimensions **(C)**. The bone-fixed local coordinate system was defined in two manners: such that the three orthonormal axes (xyz) were aligned with the XYZ axes of the global coordinate system at the zero-loading condition on the level surface (A, zero-aligned coordinate system) (Okita et al., 2014; Chen Wang et al., 2016; Ito et al., 2017); and based on the anatomical landmarks defined on the bone surfaces (B, anatomical coordinate system) (Gutekunst et al., 2013; Negishi et al., 2021). The xyz axes (blue, red, green, respectively) approximately point anteriorly, medially, and superiorly, respectively. Foot arch dimensions were (i) LA length **(C)** and (ii) LA height **(D)** and the (iii-vii) MT base height of 1–5 MTs as measures of the TA height **(E)**.

A bone-fixed local coordinate system was defined in two manners to facilitate comparisons with previous studies: 1) such that the three orthonormal axes (xyz) were aligned with the XYZ axes of the global coordinate system at the zero-loading condition on the level surface (hereafter referred to as zero-aligned bone coordinate system) (Okita et al., 2014; Chen Wang et al., 2016; Ito et al., 2017) (Figure 2A); and 2) based on the anatomical landmarks defined on the bone surfaces (hereafter referred to as the anatomical bone coordinate system) (Gutekunst et al., 2013; Negishi et al., 2021) (Figure 2B). The origin of the bone coordinate system was defined as the centroid of the corresponding bone. We quantified the changes in the positions of the bones based on their origins, and the change in the orientation of the foot bones using the y-x-z Euler angles. The x-, y-, and z-axes roughly correspond to inversion–eversion (adduction–abduction for the tibia), plantarflexion–dorsiflexion, and internal/external rotation axes. The bone-to-bone angles of the talocalcaneal (TC), talonavicular (TN), calcaneocuboid (CC), and tibiotalar (TT) joints were also calculated as the motion of the distal bone coordinate system with respect to the proximal bone coordinate system.

The 3D length and height of the longitudinal and transverse arches were calculated to quantify the deformation characteristics of the entire foot. The length of the longitudinal arch (LA length) was defined as the distance between the most inferior point of the calcaneal tuberosity and the most plantar point on the medial edge of the articular surface of the 1 MT head (Figure 2C). The height of the longitudinal arch (LA height) was defined as the perpendicular distance between the navicular tuberosity and the plantar plane defined by the most inferior point of the calcaneal tuberosity and the most plantar points on the 1 and 5 MT heads (Figure 2D). The deformation of the transverse arch (TA height) was quantified by the perpendicular distance between the midpoints of the dorsal edges of the proximal articular surfaces of the 5 MTs and the plantar plane (Figure 2E).

### Statistical Analysis

In the present study, we first evaluated the changes in the foot posture due to the coronally wedged surfaces under the zero-loading condition. Thus, the differences between the translational and rotational displacements between LS and MWS, and LS and LWS were calculated, and a one-sample t-test was performed to evaluate for statistical significance. In sequence, we assessed whether the mean translational and rotational displacements of the foot bones when the 588 N axial load was applied were significantly different from the zero-loading condition for each surface condition using paired sample t-tests. The type I error rate (alpha) was set at 0.05, but we also reported *p*-values in the results if the differences were *p* < 0.1 because the differences might have been meaningful even though the differences were not statistically significant. Statistical tests were performed using R version 4.0.4 software (R Core Team, 2021).

## Results

### Changes in the Foot Posture due to the Coronally Wedge Surfaces

**FIGURE 3:**
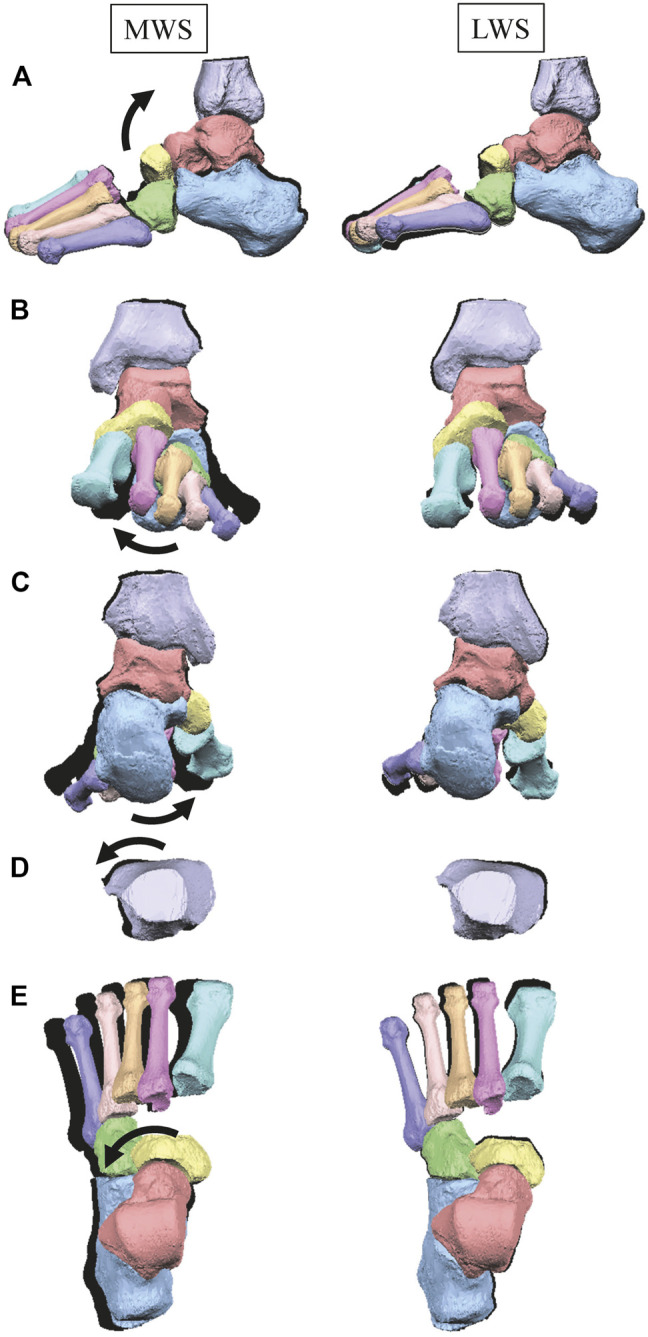
Comparisons of reconstructed three-dimensional foot posture of a representative foot on the MWS and LWS at the zero-loading condition. Lateral view **(A)**, anterior view **(B)**, posterior view **(C)**, dorsal views of the tibia **(D)**, and the tarsal bones and MTs **(E)**. The back shades indicate foot bone contours on the LS at the zero-loading condition.

**FIGURE 4:**
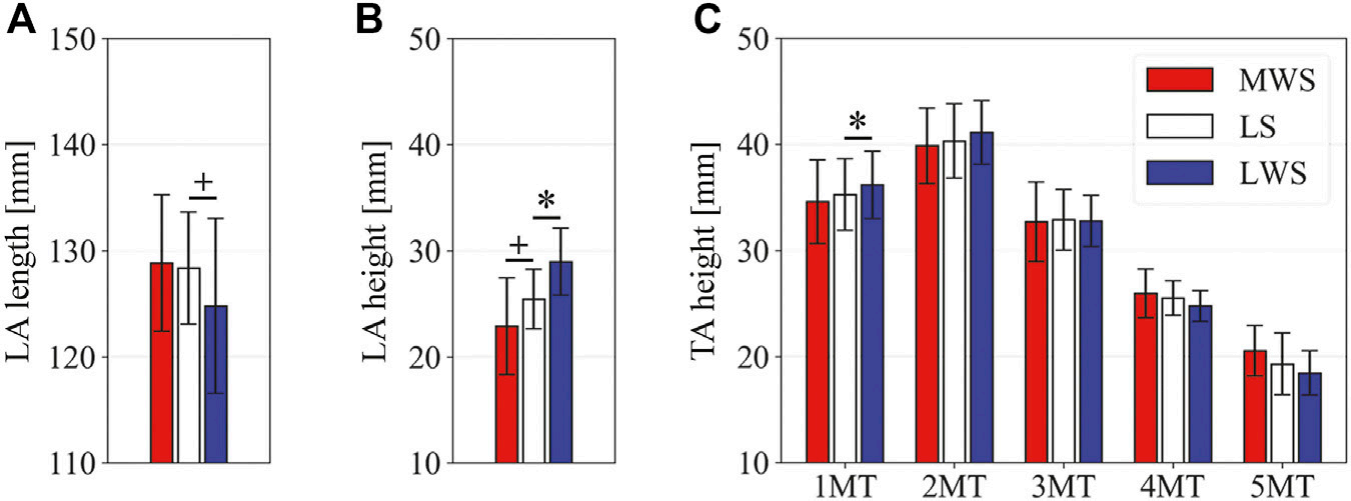
Comparisons of the foot arch dimensions on the LS, MWS, and LWS at the zero-loading condition. **(A)** LA length, **(B)** LA height, and **(C)** TA height. Error bars indicate standard deviations. *: *p* < 0.05. +: *p* < 0.1.

**FIGURE 5:**
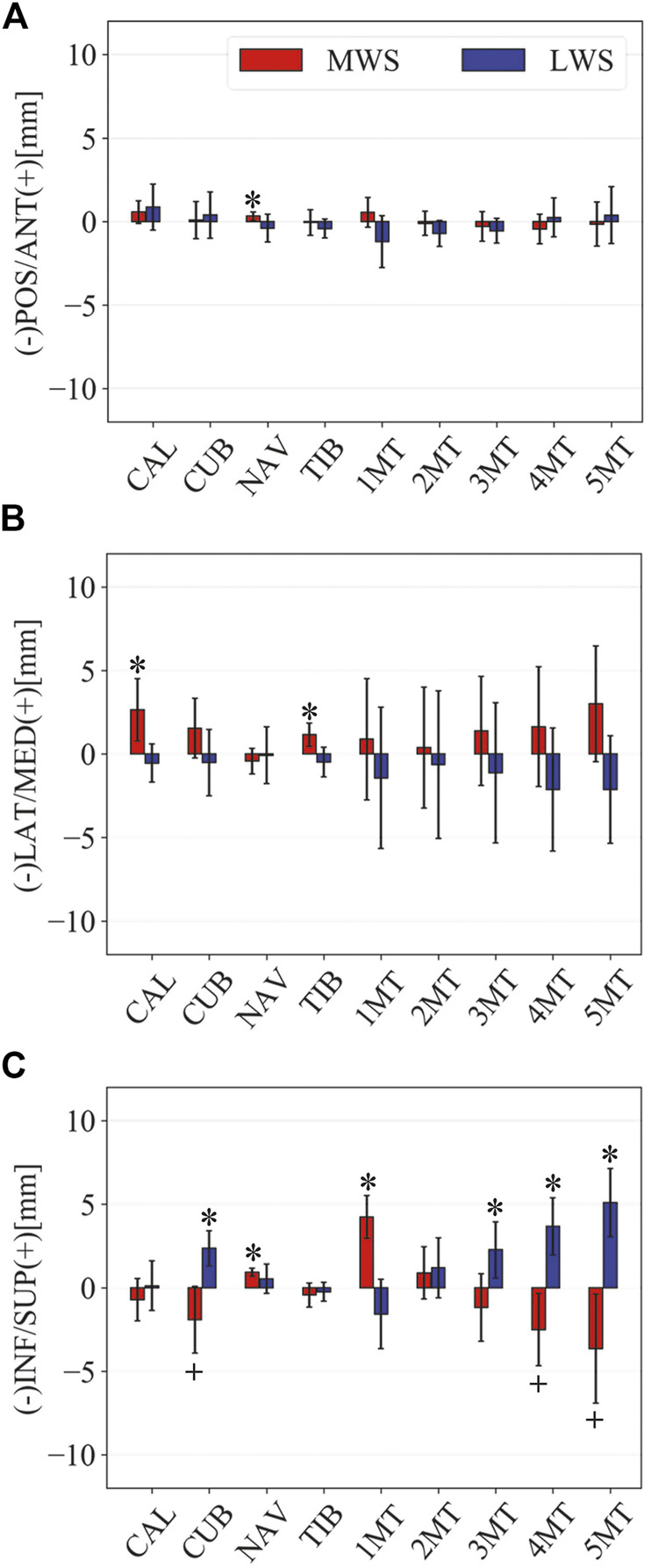
Comparisons of the changes in the position of foot bones with respect to the talus due to the wedged surfaces at the zero-loading condition. Translational displacements in the **(A)** anteroposterior, **(B)** mediolateral, and **(C)** superoinferior directions were quantified and compared. The values are positive for anterior, medial, and superior translation. Error bars indicate standard deviations. *: *p* < 0.05. +: *p* < 0.1.

**FIGURE 6:**
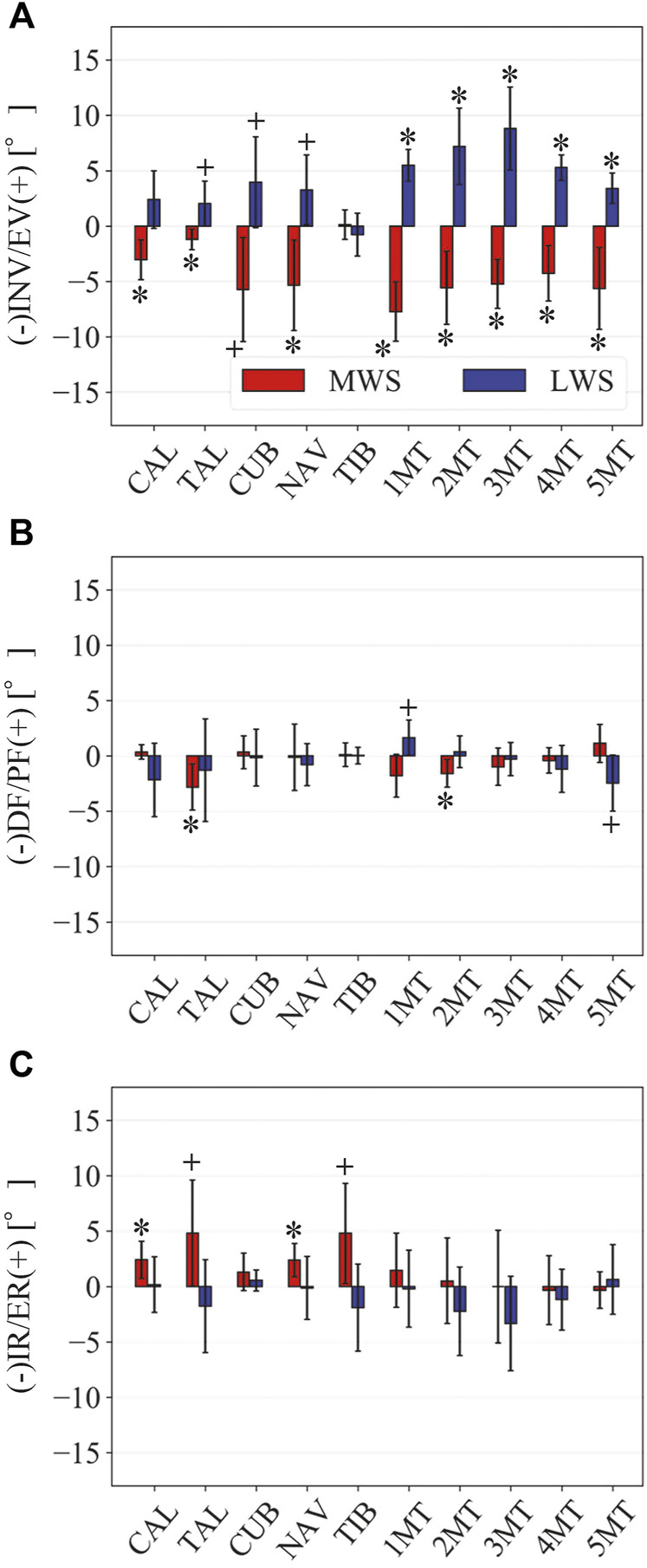
Comparisons of the changes in the orientations of the foot bones due to the wedged surface at the zero-loading condition. Rotational displacements in the **(A)** coronal, **(B)** sagittal, and **(C)** transverse planes were quantified and compared using the zero-aligned bone coordinate system. The values are positive for eversion, plantarflexion, and external rotation. Error bars indicate standard deviations. *: *p* < 0.05. +: *p* < 0.1.

**FIGURE 7:**
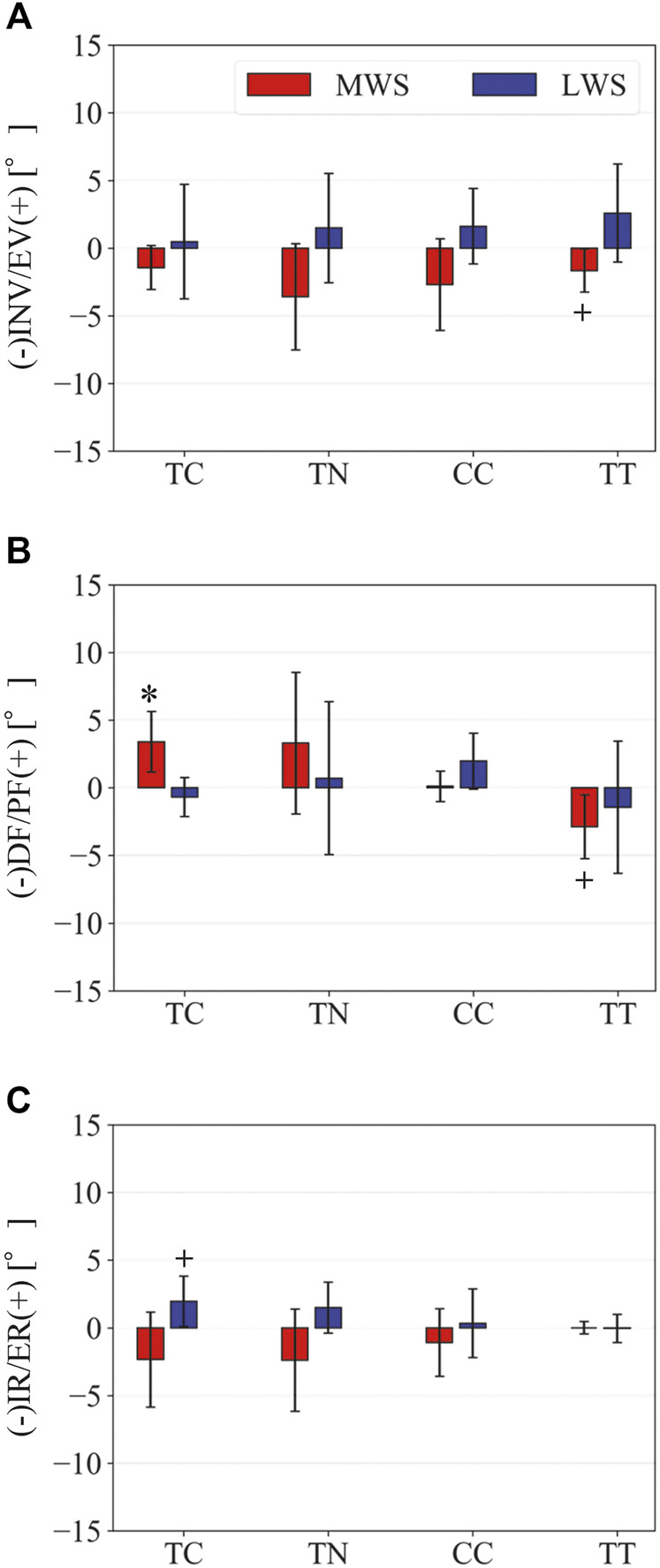
Comparisons of the changes in the TC, TN, CC, and TT joint angles due to the wedged surfaces at the zero-loading condition. **(A)** Inversion/eversion **(A)**, dorsiflexion/plantar flexion **(B)**, and internal/external rotation angles **(C)** were calculated using the zero-aligned bone coordinate system. The joint angles are positive for eversion, plantarflexion, and external rotation. Error bars indicate standard deviations. *: *p* < 0.05. +: *p* < 0.1.

Figure 3 illustrates the changes in the 3D skeletal posture of the tibia and foot bones of a representative specimen on LS, MWS, and LWS at the zero-loading condition. Figure 4 compares the foot arch dimensions on the level and wedged surfaces under the zero-loading condition. The mean LA lengths were 128.8, 128.4 and 124.8 mm and the mean LA heights were 22.9, 25.5, and 29.0 mm on the MWS, LS, and LWS, respectively, indicating that the human foot skeleton was more largely deformed (lengthened and flattened) when the foot was placed on MWS than on LS, and when the foot was placed on LS than on LWS (Figures 4A,B). The changes in the TA heights were the largest at 2 MT and decreased with increasing distance from the 2 MT, but the changes due to the surface inclination were minor (Figure 4C). Figure 5 compares the translational positions of the foot bones with respect to the talus. When the foot was placed on the MWS, the calcaneus, cuboid, and tibia translated medially with respect to the talus (2.6, 1.5, and 1.1 mm, respectively). However, when the foot was placed on the LWS, the tarsal bones did not move in the lateral direction. The MTs were translated in the medial and lateral directions when the foot was placed on the MWS and LWS, respectively. The magnitude of the MT medial and lateral movements tended to be larger with distance from the 1 MT (from 1 to 5 MT, deviations were 0.9, 0.4, 1.4, 1.6 and 3.0 mm, respectively, on the MWS, and 1.4, 0.7, 1.1, 2.1, and 2.1 mm, respectively, on the LWS; Figure 5B). The medial and lateral MTs were located superiorly and inferiorly, respectively, when the foot was placed on the MWS, and superiorly and inferiorly, respectively, when the foot was placed on the LWS, conforming to the surface orientation (Figure 5C). Figure 6 compares the changes in the foot bone orientations calculated based on the zero-aligned bone coordinate system. Conforming the surface orientation, the foot bones except for the talus and tibia generally inverted and everted when the foot was placed on the MWS and LWS, respectively (Figure 6A). The magnitudes of the inversion of the calcaneus, cuboid, and navicular were 0.6°, 1.8°, and 2.1° larger than those of eversion, although no significant differences were obtained. Horizontally, the tibia, talus, calcaneus, and navicular were externally rotated when the foot was placed on the MWS, but the opposite was not observed when the foot was on the LWS (Figure 6C). Hence the magnitude of the tibial rotation was 2.9° larger in the former than in the latter, although no significant differences were obtained. Plantarflexion/dorsiflexion movement was considerably smaller than that in the other directions (Figure 6B). Figure 7 displays the corresponding changes in joint angles. The magnitude of the inversion of the TC, TN, and CC joints on MWS was 1.0°, 2.1°, and 1.1° larger than that of the eversion on LWS, but the opposite occurred for the TT joint (0.9° smaller on the MWS than on the LWS), although no significant differences were obtained (Figure 7A). Horizontally, the TC, TN, and CC joints moved in the direction of internal rotation on the MWS and in the direction of external rotation on the LWS (Figure 7C). The magnitude of the internal/external rotation of the TT was virtually zero, indicating that the tibia was internally rotated in conjunction with the talus. The changes in the foot bone orientations and joint angles calculated based on the anatomical bone coordinate system were provided as Supplementary Information but the results were found to be broadly similar to those based on the zero-aligned bone coordinate system (Figures 6, 7).

### Foot Bone Movements Due to Axial Loading on the Coronally Wedged Surfaces

**FIGURE 8:**
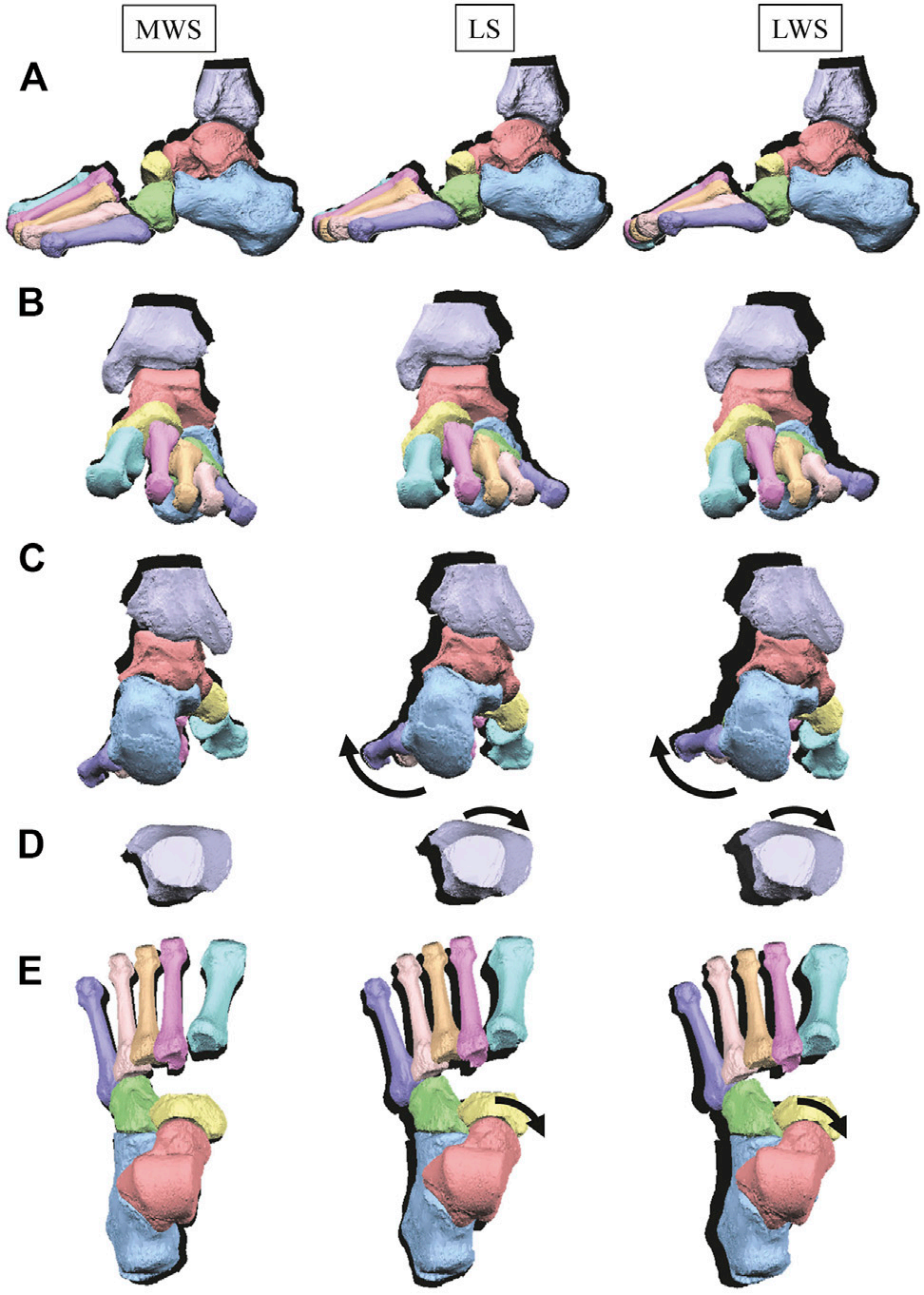
Comparisons of reconstructed three-dimensional foot bone movements of a representative foot under axial loading on the LS, MWS and LWS. Lateral view **(A)**, anterior view **(B)**, posterior view **(C)**, dorsal views of the tibia **(D)**, and the tarsal bones and MTs **(E)**. The back shades indicate foot bone contours at the zero-loading condition.

**FIGURE 9:**
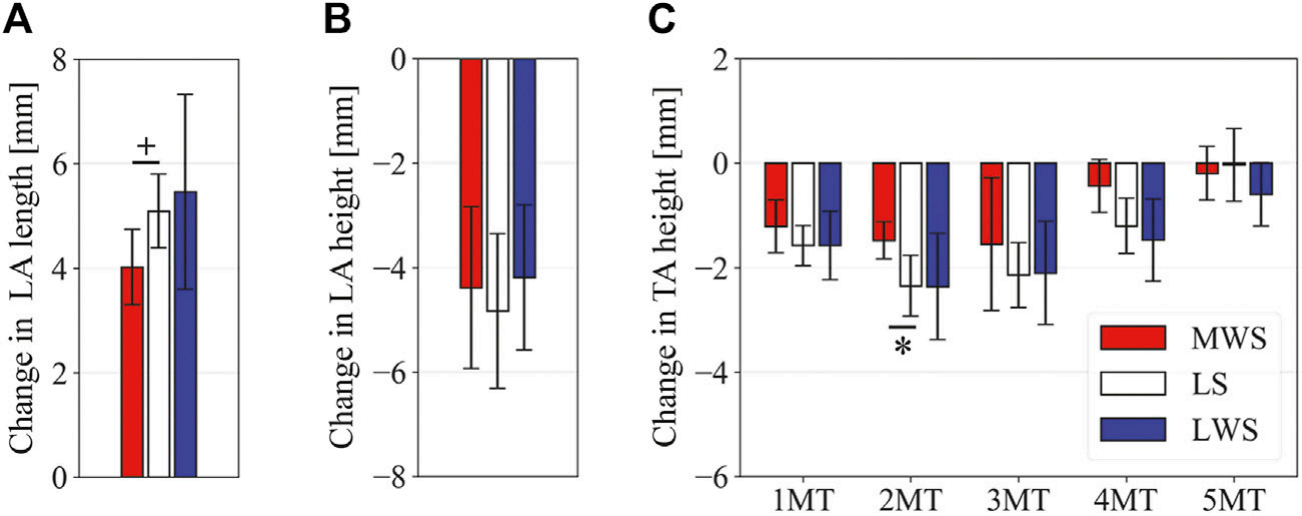
Comparisons of the changes in the foot dimensions due to axial loading on the LS, MWS, and LWS. The changes in the **(A)** LA length, **(B)** LA height, and **(C)** TA height were quantified and compared. Error bars indicate standard deviations. *: *p* < 0.05. +: *p* < 0.1.

**FIGURE 10:**
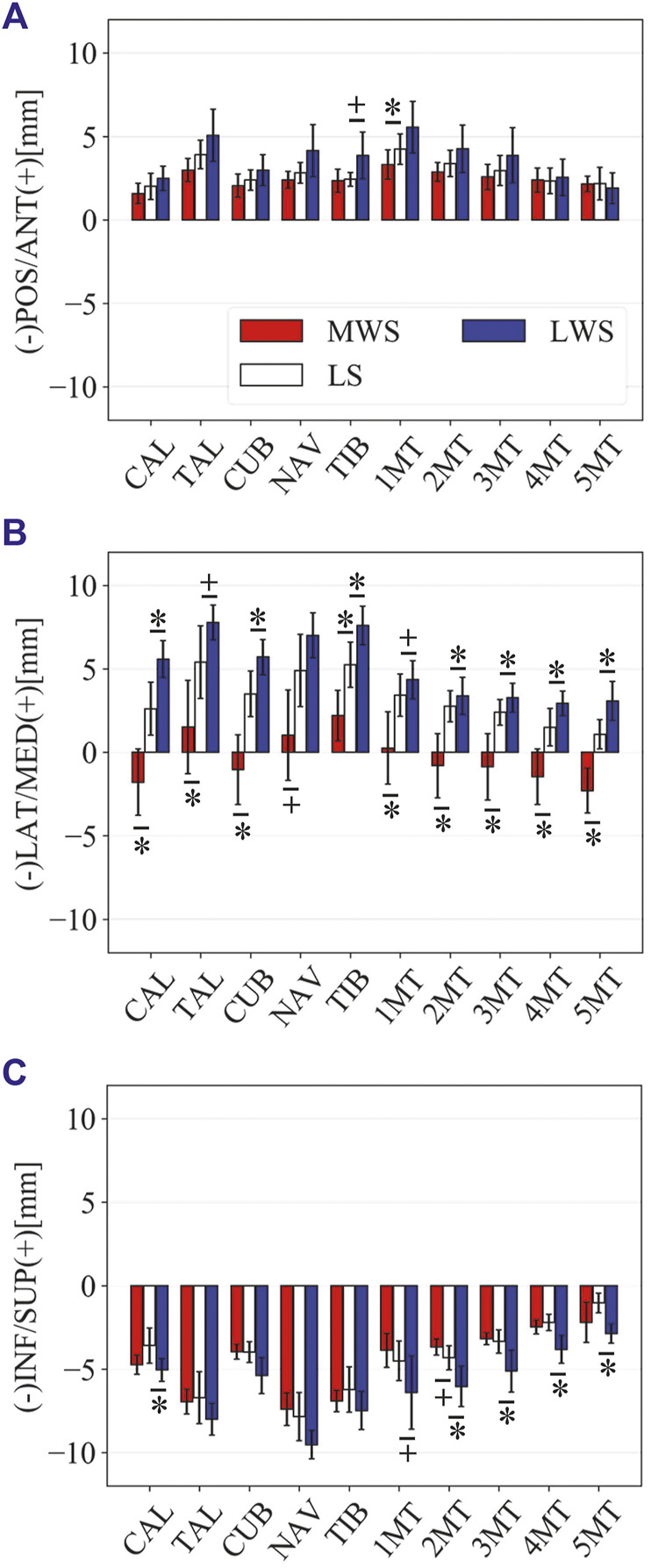
Comparisons of the changes in the position of foot bones due to axial loading on the LS, MWS, and LWS. Translational displacements in the **(A)** anteroposterior, **(B)** mediolateral, and **(C)** superoinferior directions were quantified and compared. The values are positive for anterior, medial, and superior translation. Error bars indicate standard deviations. *: *p* < 0.05. +: *p* < 0.1.

**FIGURE 11:**
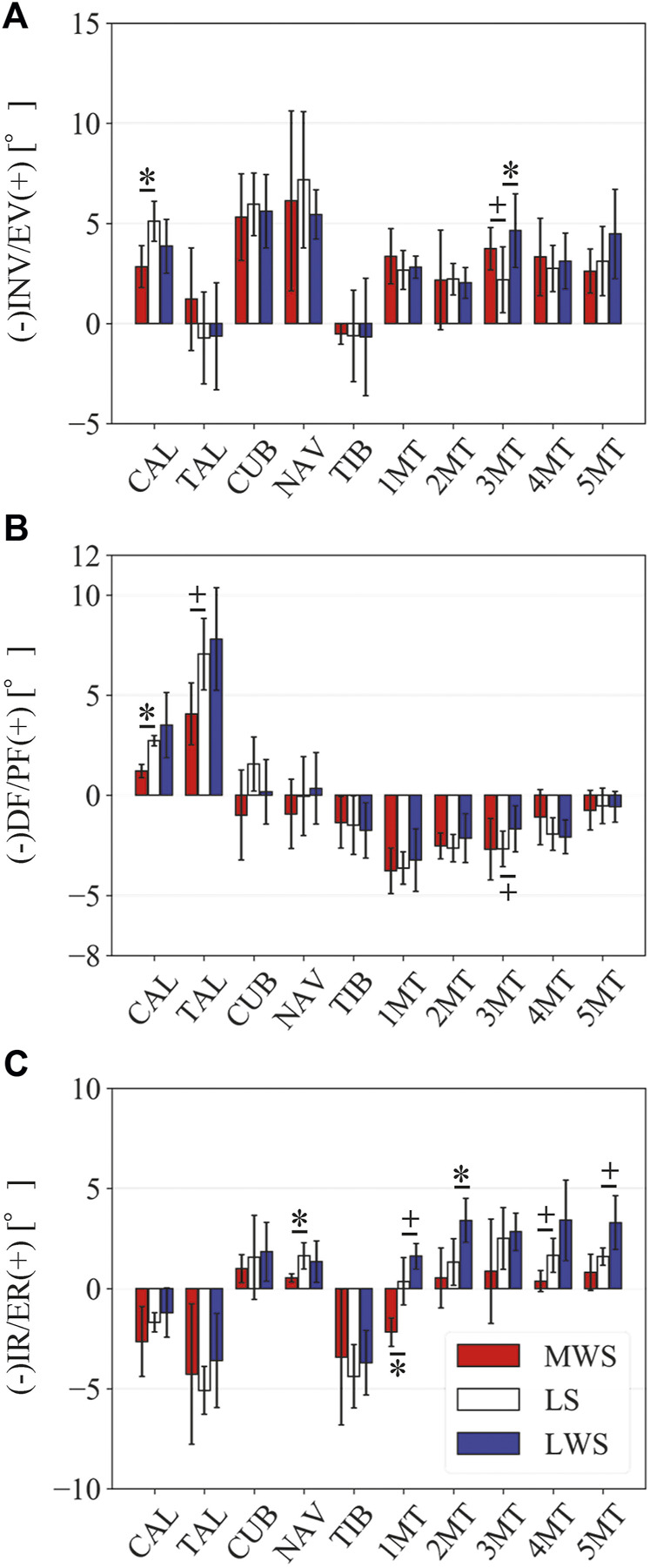
Comparisons of the changes in the orientations of the foot bones due to axial loading on the LS, MWS, and LWS. Rotational displacements in the **(A)** coronal, **(B)** sagittal, and **(C)** transverse planes were quantified and compared using the zero-aligned bone coordinate system. The values are positive for eversion, plantarflexion, and external rotation. Error bars indicate standard deviations. *: *p* < 0.05. +: *p* < 0.1.

**FIGURE 12:**
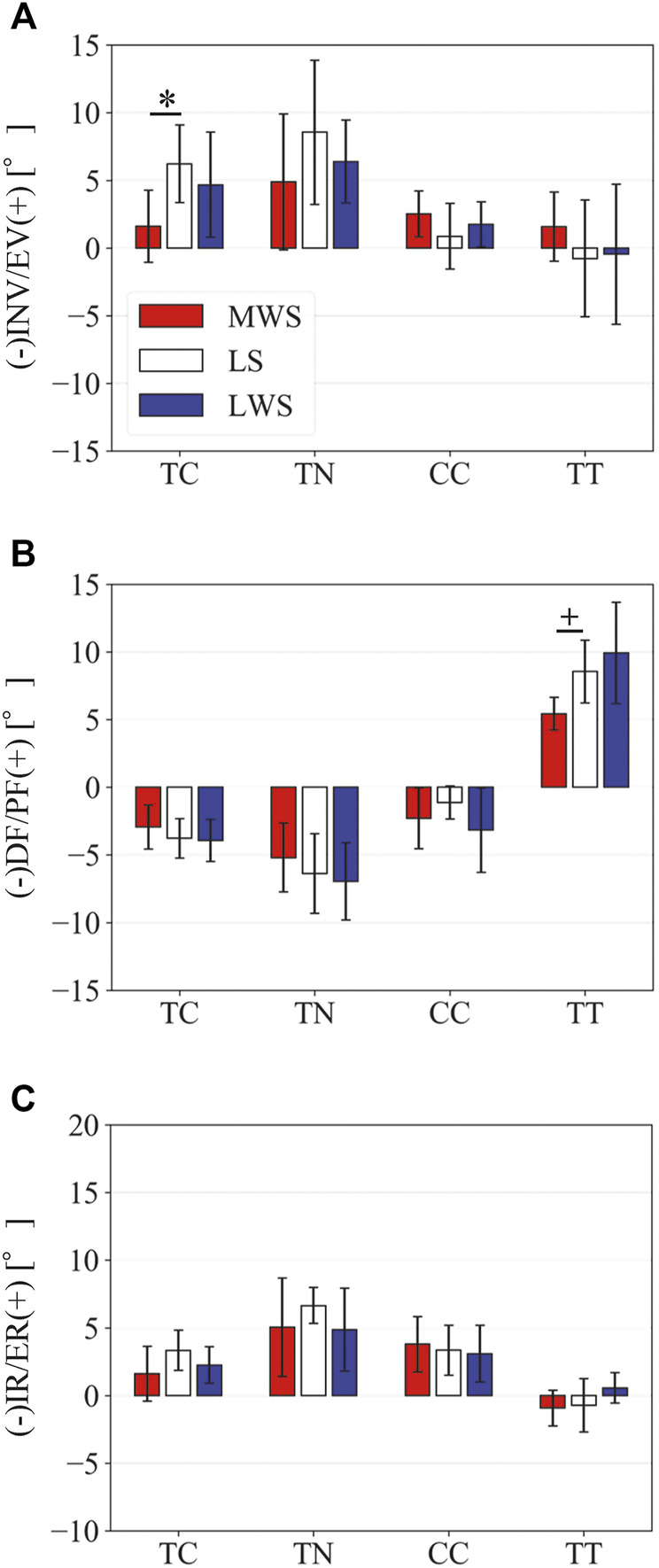
Comparisons of the changes in the TC, TN, CC, and TT joint angles due to axial loading on the LS, MWS, and LWS. **(A)** Inversion/eversion **(A)**, dorsiflexion/plantar flexion **(B)**, and internal/external rotation angles **(C)** were calculated using the zero-aligned bone coordinate system. The values are positive for eversion, plantarflexion, and external rotation. Error bars indicate standard deviations. *: *p* < 0.05. +: *p* < 0.1.

Figure 8 illustrates the 3D skeletal movements of the foot due to axial loading on the three surfaces. The foot bone movements due to axial loading on LWS were seemingly quite similar to those on LS. Figure 9 compares the changes in the foot arch dimensions of the foot on the level and coronally wedged surfaces during axial loading. The changes in the LA length were 4.0, 5.1, and 5.5 mm on the MWS, LS, and LWS, respectively, and the changes in the TA height from 1 to 5 MT were larger in LWS (−1.6, −2.4, −2.1, −1.5, and −0.6 mm, respectively) and LS (−1.6, −2.4, −2.1, −1.2, and 0.0 mm, respectively) than in MWS (−1.2, −1.5, −1.6, −0.4, and −0.2 mm, respectively) (Figures 9A,C), suggesting that the foot on LWS or LS was more compliant than that on MWS when axially loaded, and indicating that the foot was more rigid if it was inverted. Figure 10 shows a comparison of the translational movements of the foot bones due to axial loading. The foot bones generally translated anteriorly and inferiorly (Figures 10A,C). The inferior translations of the talus, cuboid, navicular, and 5 MTs were 1.1 mm (*p* = 0.038), 1.4 mm (*p* = 0.022), 2.1 mm (*p* = 0.015), 2.5 mm (*p* = 0.023), 2.4 mm (*p* = 0.009), 1.9 mm (*p* = 0.030), 1.4 mm (*p* = 0.045) and 0.7 mm (n.s.) larger on the LWS than on the MWS. On LS and LWS, the foot bones translated laterally with axial loading, whereas they translated slightly medially when the foot was placed on the MWS (Figure 10B). Figure 11 compares the changes in the orientation of the bones due to axial loading calculated based on the zero-aligned coordinate system. The corresponding graphs based on the anatomical bone coordinate system were provided as Supplementary Information, but the results were broadly similar. Owing to axial loading, the foot bones were generally everted, except for the talus and tibia (Figure 11A). The calcaneus and talus were plantarflexed and internally rotated, whereas the MTs were dorsiflexed and externally rotated (Figures 11B,C). Along with the talus, the tibia was internally rotated due to axial loading. No clear differences in the bone orientations due to axial loading were observed among the three surface conditions; however, eversion of the calcaneus was more restricted on MWS (2.3° smaller on MWS than LS, *p* = 0.038; Figure 11A). Figure 12 displays the changes in the joint angles owing to axial loading. The TC and TN joints were everted, dorsiflexed, and externally rotated for all three cases, but the eversion of the TC joint was relatively more restricted on MWS (4.6° smaller on MWS than LS, *p* = 0.019; Figure 12A). The TT was plantarflexed in all cases.

## Discussion

The human foot can be inverted and everted when standing and walking on coronally wedged surfaces to maintain balance. The present study demonstrated that the human foot was more largely deformed from the natural posture to conform to the coronally inclined surface when the foot was placed on MWS than on LWS (Figures 4–7). In the present study, the tibia was oriented vertically in all cases, and the wedge angle was identical for both the MWS and LWS. However, the relative contributions of the number of joint rotations were different between the two cases. When the foot was inverted due to MWS, the rotational displacements of the TC, TN, and CC were relatively larger, and that of the TT was smaller than when the foot was everted due to LWS (Figure 7). Conversely, when the foot was everted due to LWS, the rotational displacements of the TC, TN, and CC were relatively smaller, and that of the TT was larger than when the foot was inverted due to MWS. This possibly suggests that the human foot is more capable of rotating in the inverting direction, but the rotation in the everting direction is somewhat restricted, possibly because of the mobility of the TC, TN, and CC joints determined by the bony structure of the articular surfaces of these joints. The range of motion of the human ankle joint complex in the eversion-inversion direction is reportedly larger in the latter (Allinger and Engsberg, 1993), indicating that the ankle joint is more mobile and hence less stable in the inverting direction; thus, there is a higher chance for the ankle to be excessively inverted after foot contact on a wedged surface in activities such as gait and jumping. This innate mobility of the ankle joint complex partly explains why lateral ankle sprains were more common than medial sprains (Woods et al., 2003; Swenson et al., 2013). The present study showed some of the structurally embedded mechanisms underlying the innate mobility of the human foot.

The fact that the human foot is more mobile in the inverting direction could possibly be linked to the evolutionary history of the human foot. Molecular studies of African great apes have indicated that chimpanzees, bonobos, and gorillas are the closest living taxa of humans (Kuhlwilm et al., 2016). Based on parsimony, the last common ancestor of humans and chimpanzees may have possessed inverted feet, as in African great apes. During the process of human evolution, the human may have retained the ability to more flexibly invert the foot to better conform to MWS, possibly because this ability is adaptive even on the ground when the feet need to be laterally spread for stability, although the shape of the trochlea of the talus was altered from conical to cylindrical to place the foot more perpendicularly with respect to the tibia to adapt to terrestrial locomotion (Latimer et al., 1987). The ability to evert the foot was probably less important because the legs certainly stumble into one another when trying to cross the legs in the coronal plane; therefore, the ability to flexibly evert was less practical.

The present study also demonstrated that the tibia was externally rotated when the foot was placed on the MWS due to the inversion of the calcaneus with respect to the global coordinate system, and internally rotated when the foot was placed on the LWS due to the eversion of the calcaneus (Figures 6, 7). The magnitude of the tibial rotation was larger in the former than in the latter. These observations are consistent with previous cadaver studies documenting the kinematics of the tibio-calcaneal coupling (Lapidus, 1963; Olerud and Rosendahl, 1987; Hintermann et al., 1994). However, these studies did not observe how the talus moved due to the tibio-calcaneal coupling because motion measurement of the talus was difficult based on conventional methods. Using a biplane fluoroscopic system, the present study demonstrated that the talus is externally and internally rotated when the calcaneus is inverted and everted, respectively, and the external/internal rotation is kinematically transmitted to the tibia. This is because the bones forming the TT joint, tibia, fibula, and talus are interlocked with one another, allowing firm transmission of the internal/external rotation of the talus to that of the tibia. In a recent study, the coupling movement of the inversion/eversion of the calcaneus and the external/internal rotation of the tibia was reported to be accompanied by abduction/adduction (external/internal rotation) of the calcaneus (Fischer et al., 2018). This tendency was also confirmed in the present study (Figure 6).

The present study was comparable to the *in vivo* X-ray study by Lundberg et al. (1989) on the 3D kinematics of the human foot when placed on coronally inclined surfaces. The results are also generally consistent with those reported in the literature. However, the patterns of inversion/eversion and dorsiflexion/plantarflexion of the TT joint were different from each other (Figure 7). This is possibly due to the difference in the manner the tibia was placed in the global coordinate system. In our *in vitro* cadaver experiment, the tibia was always vertically oriented by the shaft fixed to the tibia and a linear guide attached to the apparatus (Figure 1); however, in the *in vivo* experiment by Lundberg et al. (1989), the orientation of the lower leg with respect to the foot (talus) was not constant but could be altered because the participants had to maintain balance on a single leg while standing on a coronally inclined surface that was varying. Therefore, we believe that the experimental conditions were better controlled in the present study, leading to a more precise understanding of the innate foot bone movements that are inherently prescribed in the structure of the human foot.

The pattern of the foot bone translations and rotations due to axial loading did not significantly differ among the three surface conditions; the bones were anteriorly and inferiorly translated, and generally everted except for the talus and tibia (Figures 10, 11). However, the present study indicated that the eversion of the calcaneus due to axial loading was relatively more restricted (Figures 11, 12). This may explain why the degree of flattening of the foot due to axial loading was relatively larger for LWS and LS than for MWS (Figure 9). If the foot is inverted, the calcaneal tuberosity is pointed more medially, hindering eversion of the calcaneus because the horizontal distance between the calcaneal tuberosity and the talus is shortened (Negishi et al., 2021). Therefore, the foot on the MWS was less deformable owing to axial loading.

Previous studies have attempted to clarify the effects of lateral or medial wedge insoles on the kinematics and dynamics of human gait. These studies demonstrated that the calcaneus and tibia were less everted and internally rotated in the early stance phase when wearing a medial wedge insole compared to the control condition (Mündermann et al., 2003; MacLean et al., 2006; Eslami et al., 2009). The same tendency was observed during walking in experimental shoes with a varus wedge (Lafortune et al., 1994). These observations corroborate the present results, indicating that the human foot on MWS was less everted and internally rotated than that on LS and LWS during axial loading (Figure 11). The present study possibly supports the clinical utility of the medial wedge insole on aching pain associated with flatfoot deformity (Braga et al., 2019) and ligamentous sprain of the anterior talofibular ligament (Tochigi, 2003). Conversely, when wearing a lateral wedge insole, the calcaneus was only slightly everted (Kakihana et al., 2005; Butler et al., 2009; Hatfield et al., 2016) compared to the control condition. This is also consistent with the present results indicating that the magnitudes of the calcaneal eversion were not different between LWS and LS (Figure 11); the LWS does not necessarily induce eversion of the calcaneus due to the restricted range of the TC joint in the everting direction. However, this might facilitate control of walking gait by providing stability of the stance foot in the coronal plane in patients with knee osteoarthritis. It must be noted, however, that the findings of the present study must be carefully translated *in vivo* since they are based on the measurements *in vitro*.

One limitation of the present study was that the cadaver feet were only from elderly individuals, and the elastic properties of the plantar soft tissue and joint mobility are known to change with aging (James and Parker, 1989; Morag and Cavanagh, 1999). Therefore, the results might differ by using cadavers from young individuals, which are rarely available. Another limitation is the small sample size in the present study as it reduces the power of the present study. However, the availability of cadaver specimens is generally very limited, and the sample size of five is the same as that in other similar cadaver studies (e.g., Tochigi, 2003; Ito et al., 2017). Although we believe that the current study successfully extracted the structurally embedded basic pattern of how the human foot bones move on coronally inclined surfaces, the extracted findings must be confirmed with a large sample size when such an opportunity arises. For the same reason, the present study did not consider possible gender differences in the innate mobility of the foot, although previous studies have suggested that there exists sexual dimorphism in the morphology of the foot between females and males (Ferrari et al., 2004; Moore et al., 2019; Tümer et al., 2019; Nozaki et al., 2020a; Nozaki et al., 2020b) that possibly leads to differences in the kinematics of the foot during walking (Fukano and Fukubayashi, 2012; Lee et al., 2016; Takabayashi et al., 2017; Fukano et al., 2018). Although there is in fact a large overlap in the distribution of the foot morphology and kinematics between genders, this is also a limitation of the present study. The last limitation is that we did not investigate the effects of the flexion/extension angle of the ankle joint on the mobility of the foot bones placed on the coronally wedged surfaces, although the ankle joint angle supposedly affects the foot bone mobility (Hintermann et al., 1994). This must also be investigated in future studies.

## Conclusion

The present study provided new insights about the kinematic adaptation of the foot on coronally wedged surfaces that is inherently embedded and prescribed in the anatomical structure of the human foot by quantifying 3D entire foot bone movements. Specifically, we found that the human foot was more largely deformed from the natural posture when the foot was placed on the MWS than on the LWS. Moreover, the talus and tibia were externally rotated when the foot was placed on the MWS due to the inversion of the calcaneus, and they were internally rotated when the foot was placed on the LWS due to the eversion of the calcaneus, owing to the structurally embedded mobility of the human talocalcaneal joint. Deformation of the foot during axial loading was relatively smaller on the MWS due to restricted eversion of the calcaneus. Such descriptions will contribute to interpreting the functional adaptation of the human foot to bipedal walking and also to understanding the pathogenetic mechanism or improving the treatment and prevention of foot and lower leg injuries. In future, how the morphologically embedded mobility of the human foot extracted in the present study could actually contribute to the mechanics of human bipedal locomotion *in vivo* should be investigated as the present cadaver study did not consider the contributions of foot muscles activations which possibly alter the mechanical interaction of the feet with the ground. In addition, future research should focus on the translation of the current findings to possible clinical applications, such as development of insoles to reduce risk and pain of foot pathologies as well as improvement of total ankle arthroplasty.

## Data Availability

The original contributions presented in the study are included in the article/Supplementary Material, further inquiries can be directed to the corresponding authors.
