# Supplementary material for: Three-Dimensional Innate Mobility of the Human Foot on Coronally-Wedged Surfaces Using a Biplane X-Ray Fluoroscopy
Source: Front Bioeng Biotechnol. 2022 Feb 4;10:800572. doi: 10.3389/fbioe.2022.800572 (PMC8854865; doi:10.3389/fbioe.2022.800572)
Supplement: Supplementary file 1 [file DataSheet2.docx]

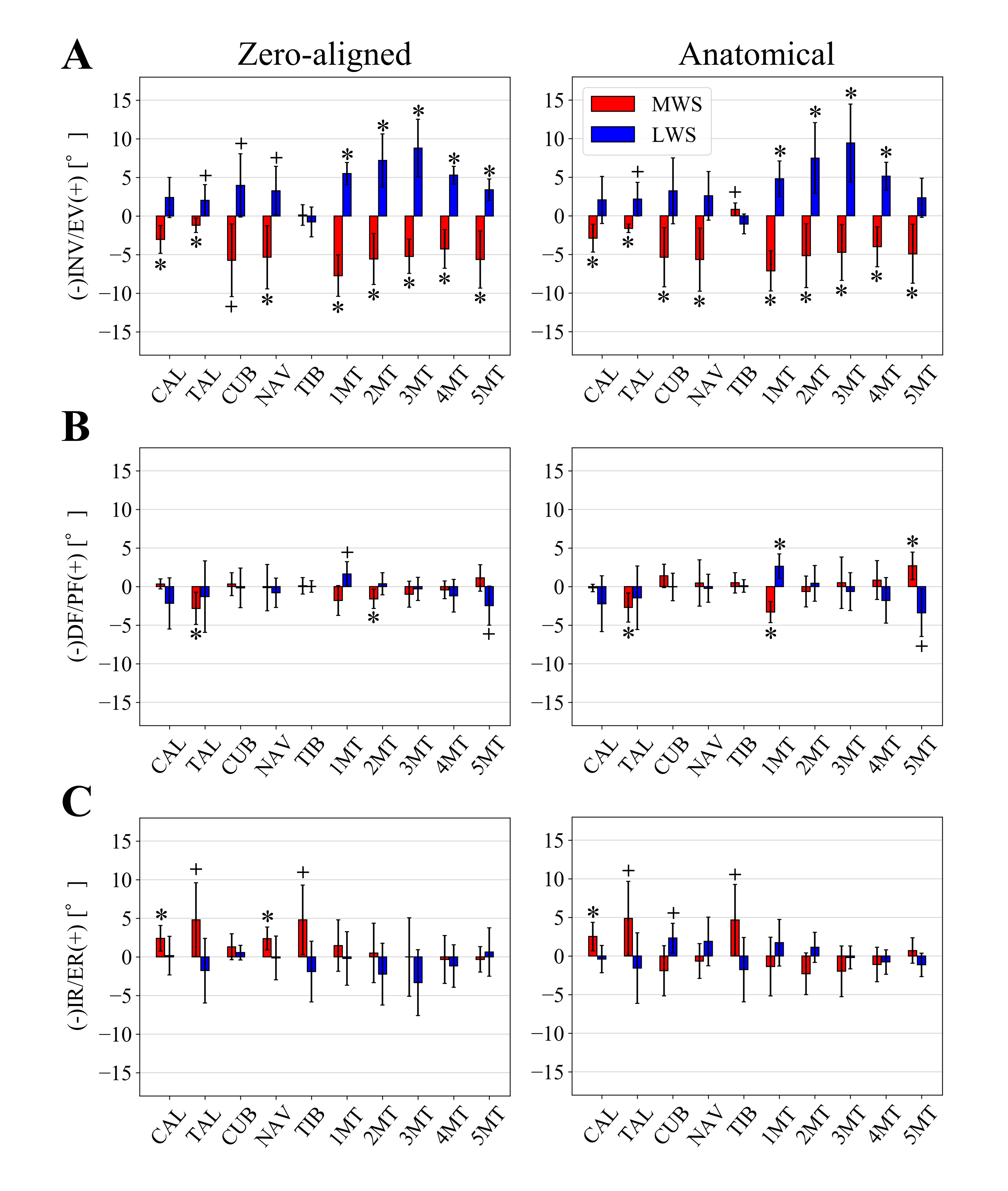


**Figure S1.** Comparisons of the changes in the orientations of the foot bones due to the wedged surface at the zero-loading condition. Rotational displacements in the (A) coronal, (B) sagittal, and (C) transverse planes were quantified and compared using the zero-aligned and anatomical bone coordinate systems. The values are positive for eversion, plantarflexion, and external rotation. Error bars indicate standard deviations. *: *p* < 0.05. +: *p* < 0.1.


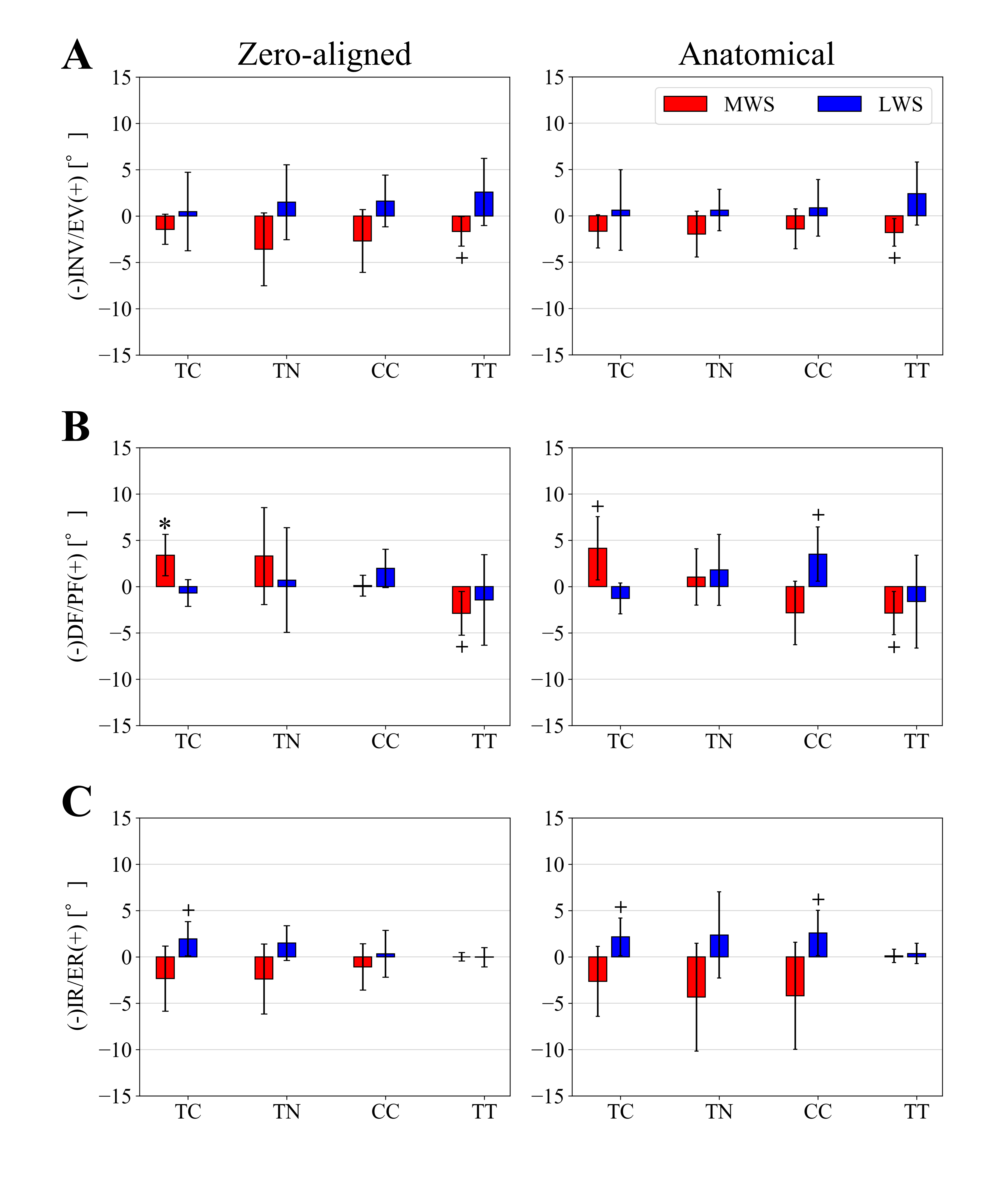


**Figure S2.** Comparisons of the changes in the TC, TN, CC, and TT joint angles due to the wedged surfaces at the zero-loading condition. (A) Inversion/eversion (A), dorsiflexion/plantar flexion (B), and internal/external rotation angles (C) were calculated using the zero-aligned and anatomical bone coordinate systems. The joint angles are positive for eversion, plantarflexion, and external rotation. Error bars indicate standard deviations. *: *p* < 0.05. +: *p* < 0.1.


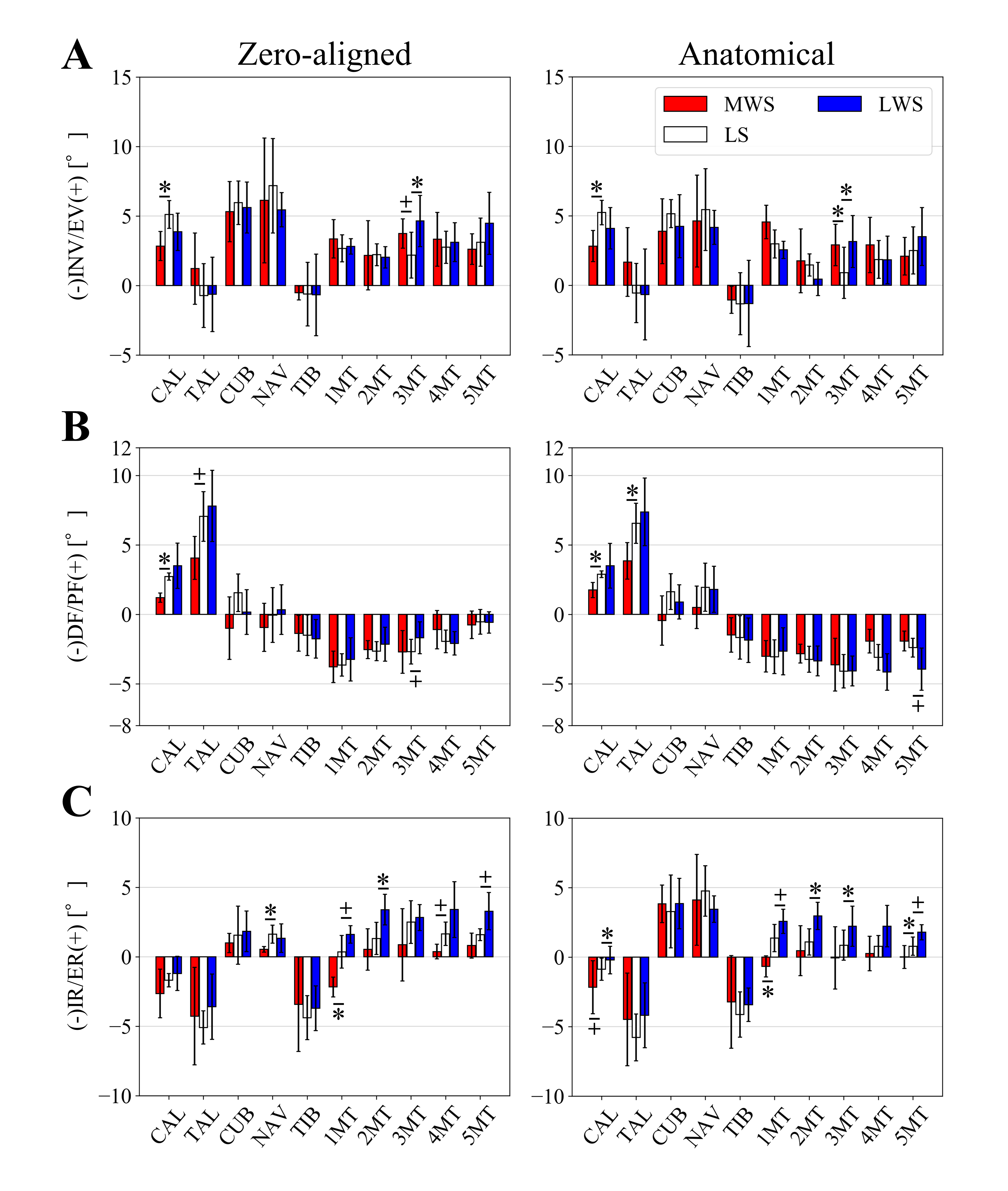


**Figure S3.** Comparisons of the changes in the orientations of the foot bones due to axial loading on the LS, MWS, and LWS. Rotational displacements in the (A) coronal, (B) sagittal, and (C) transverse planes were quantified and compared using the zero-aligned and anatomical bone coordinate systems. The values are positive for eversion, plantarflexion, and external rotation. Error bars indicate standard deviations. *: *p* < 0.05. +: *p* < 0.1.


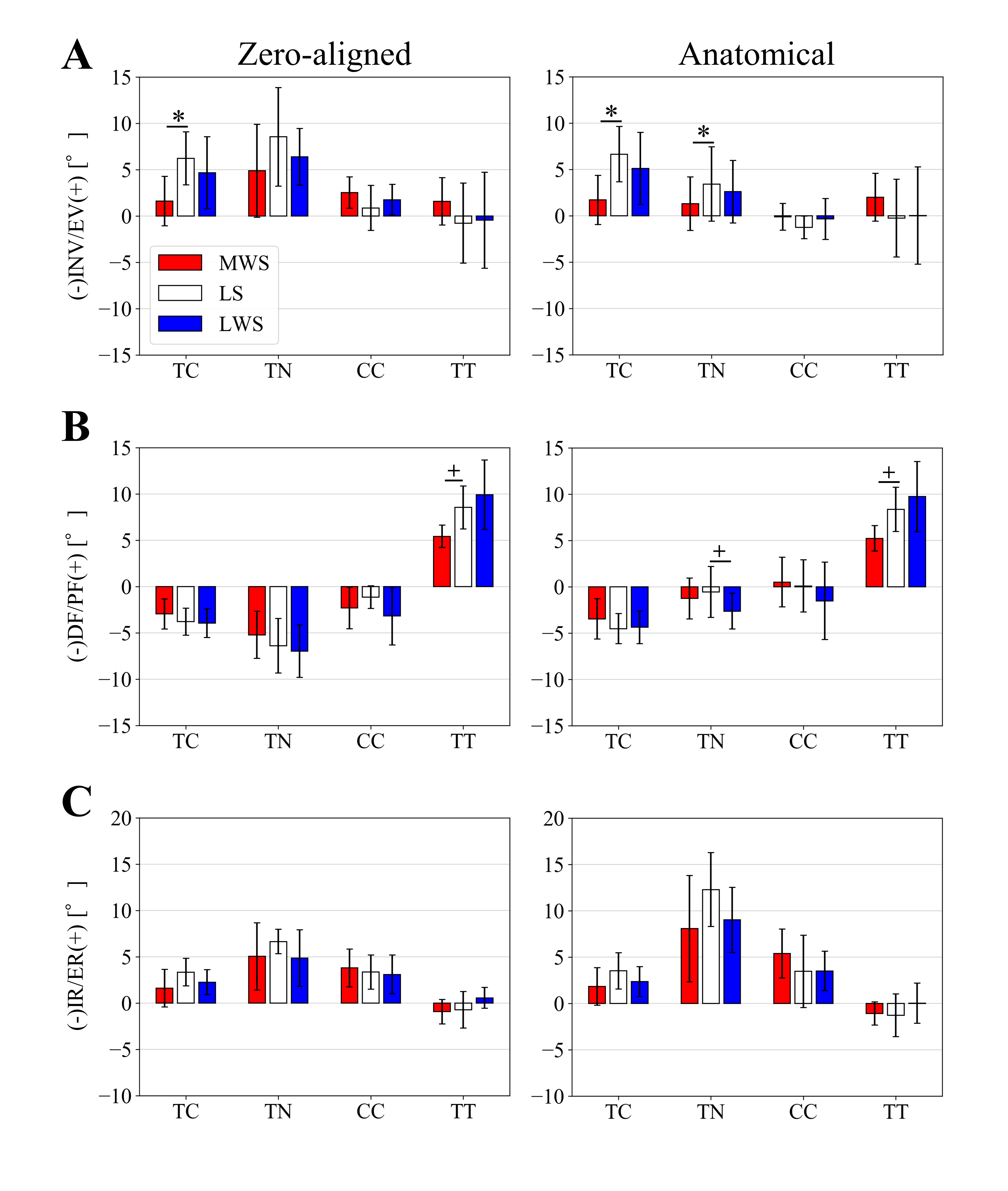


**Figure S4.** Comparisons of the changes in the TC, TN, CC, and TT joint angles due to axial loading on the LS, MWS, and LWS. (A) Inversion/eversion (A), dorsiflexion/plantar flexion (B), and internal/external rotation angles (C) were calculated using the zero-aligned and anatomical bone coordinate systems. The values are positive for eversion, plantarflexion, and external rotation. Error bars indicate standard deviations. *: *p* < 0.05. +: *p* < 0.1.
